# Supplementary material for: Kitaev interactions through extended superexchange pathways in the jeff = 1/2 Ru3+ honeycomb magnet RuP3SiO11
Source: Nat Commun. 2024 Nov 15;15:9778. doi: 10.1038/s41467-024-53900-3 (PMC11568271; doi:10.1038/s41467-024-53900-3)
Supplement: Supplementary file 1 — Supplementary Information [file 41467_2024_53900_MOESM1_ESM.pdf]

Supplementary Information: Kitaev Interactions Through Extended Superexchange Pathways in the $j_{\mathsf{eff}} = 1/2$ Ru^{3+} Honeycomb Magnet RuP_3SiO_{11}

Aly H. Abdeldaim[1,2,3*], Hlynur Gretarsson[4], Sarah J. Day[3], M. Duc Le[2], Gavin B. G. Stenning[2], Pascal Manuel[2], Robin S. Perry[2,5], Alexander A. Tsirlin[6], Gøran J. Nilsen[2*], Lucy Clark[1*]

[1]School of Chemistry, University of Birmingham, Edgbaston, Birmingham, B15 2TT, UK.
[2]ISIS Neutron and Muon Source, Didcot, Oxfordshire, OX11 0QX,UK.
[3]Diamond Light Source, Didcot, Oxfordshire, OX11 0DE, UK.
[4]Deutsches Elektronen-Synchrotron DESY, Hamburg, D-22607, Germany.
[5]London Centre for Nanotechnology and Department of Physics and Astronomy, University College London, London, WC1E 6BT, UK.
[6]Felix Bloch Institute for Solid-State Physics, University of Leipzig, Leipzig, 04103, Germany.

*Corresponding author(s). E-mail(s): aly.abdeldaim@diamond.ac.uk; goran.nilsen@stfc.ac.uk; l.m.clark@bham.ac.uk;

Keywords: Quantum materials, Kitaev quantum spin liquid, materials synthesis, neutron scattering, X-ray scattering.

Supplementary Notes

The supplementary notes contain further details on the crystal structure refinement of RPSO, field-dependent magnetic susceptibility and isothermal magnetisation measurements, supporting density-functional theory calculations and inelastic neutron scattering analysis.

Supplementary Note 1: Crystal Structure Refinement

The $R\bar{3}c$ structural model of RuP_3SiO_{11} was confirmed through Rietveld analysis of high-resolution synchrotron X-ray and neutron powder diffraction at temperatures of 300 K, 2.5 K, and 0.08 K (see Supplementary Figure 1). The temperature-dependent analysis, detailed in Supplementary Tables. 1-2, shows an isotropic contraction of the unit cell parameters that are consistent with the isotypic compounds, MoP_3SiO_{11} [1] and FeP_3SiO_{11} [2].

Supplementary Note 2: Temperature-Field Magnetic Phase Diagram

As shown in Supplementary Figure 2a, the magnetization isotherm of RPSO measured at 0.4 K, (below $T_{\mathsf{N}} = 1.3$ K), exhibits an anomaly at a critical field of $H_{\mathsf{C}} = 3.55$ T. The temperature dependence of this anomaly is displayed in Supplementary Figure 2b, which softens as the paramagnetic regime is approached. A closer examination of the field and temperature dependence, shown in Supplementary Figure 2c, of M/H reveals that beyond 0.1 T, the anomaly indicating the onset of long-range magnetic order broadens, and its temperature dependence decreases steadily with increasing applied field. Upon reaching 3.5 T, T_{N} is no longer discernible within the $0.5 - 1.7$ K temperature range of the measurement.

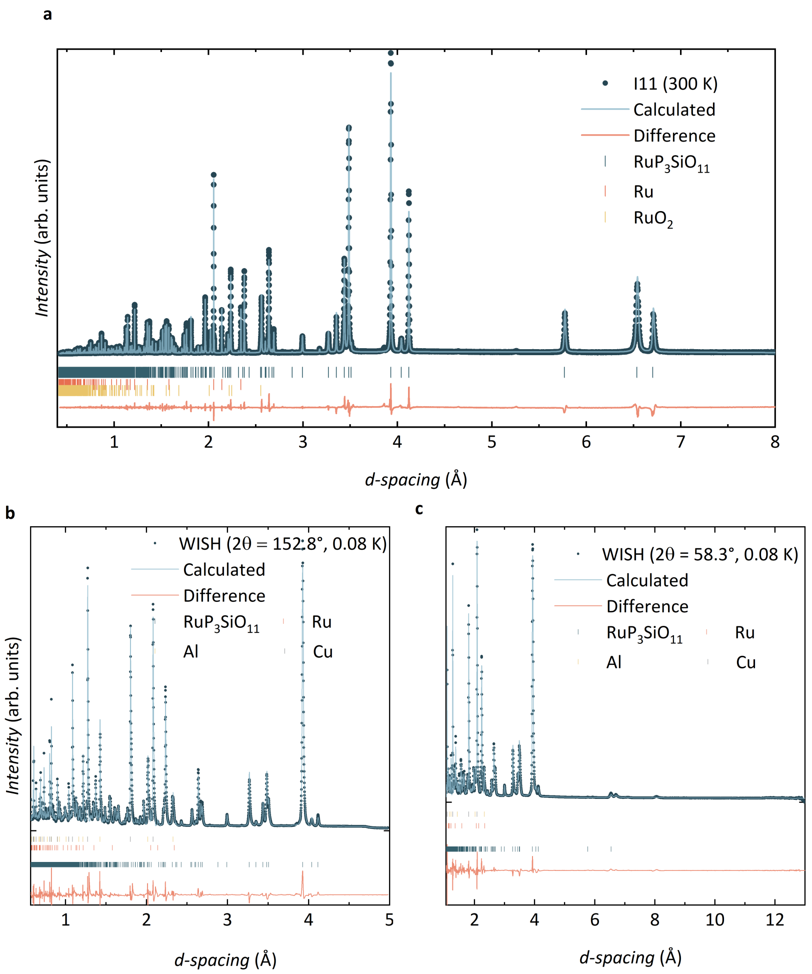

Supplementary Figure 1 **a** Rietveld analysis of powder X-ray diffraction data (300 K, I11, Diamond) using the $R\bar{3}m$ structural model that describes the crystal structure of RuP_3SiO_{11}. **b,c** Rietveld analysis of powder neutron diffraction data (WISH, ISIS) confirming that RuP_3SiO_{11} maintains the $R\bar{3}c$ structural model down to a base temperature of 0.08 K.

Supplementary Table 1 Structural parameters of RuP_3SiO_{11} obtained by Rietveld refinement of the $R\bar{3}c$ model against powder X-ray diffraction data measured at 300 K on I11 (Diamond). The resulting unit cell parameters are $a = b = 8.2410(1)$ Å and $c = 39.230(1)$ Å with goodness-of-fit parameters $\chi^2 = 3.55$ and $R_\mathrm{p} = 8.06\%$.

Atom	Site	x	y	z	U_iso (Å^2)
Ru	12c	0	0	0.15854(1)	0.0057(1)
P	36f	0.3707(1)	0.0377(1)	0.11972(1)	0.0003(2)
Si	12c	0	0	0.04017(4)	0.0012(1)
O1	36f	0.0440(3)	0.1929(3)	0.05235(5)	0.0037(6)
O2	36f	0.2233(3)	0.0829(2)	0.12706(5)	0.0043(5)
O3	36f	0.1374(3)	0.2322(3)	0.18896(5)	0.0097(6)
O4	18e	0.2101(3)	0	$\frac{3}{4}$	0.0027(4)
O5	6b	0	0	0	0.0110(7)

Supplementary Note 3: First-Principles Calculations

Ab initio methods were used to independently verify the validity of the $j_\mathrm{eff} = \frac{1}{2}$ character of Ru^{3+} in RPSO as determined in the main manuscript by RIXS, as well as to investigate the nature of the exchange Hamiltonian. Density-functional theory (DFT) band structure calculations were performed in the FPLO [3] and VASP [4, 5] codes using the experimentally determined crystal structure and the Perdew-Burke-Ernzerhof (PBE) [6] flavour of the exchange-correlation potential. Correlation effects in the Ru 4d shell were accounted for on the mean-field DFT+U level with

Supplementary Table 2 Structural parameters of RuP_3SiO_{11} obtained by Rietveld refinement of the $R\bar{3}c$ model against powder neutron diffraction data measured at 2.5 K on WISH (ISIS). The resulting unit cell parameters are $a = b = 8.2297(5)$ Å and $c = 39.20(2)$ Å with goodness-of-fit parameters $\chi^2 = 3.23$ and $R_\mathrm{p} = 5.97\%$.

Atom	Site	x	y	z	U_iso (Å^2)
Ru	12c	0	0	0.1583(2)	0.02(1)
P	36f	0.3720(8)	0.0381(8)	0.1196(1)	0.02(1)
Si	12c	0	0	0.0397 (3)	0.001(2)
O1	36f	0.0484(7)	0.2042(6)	0.0533(1)	0.02(3)
O2	36f	0.2273(7)	0.0877(7)	0.1273(1)	0.01(2)
O3	36f	0.1377(6)	0.2316(6)	0.1891(1)	0.009(3)
O4	18e	0.2080(8)	0	$\frac{3}{4}$	0.01(1)
O5	6b	0	0	0	0.02(3)

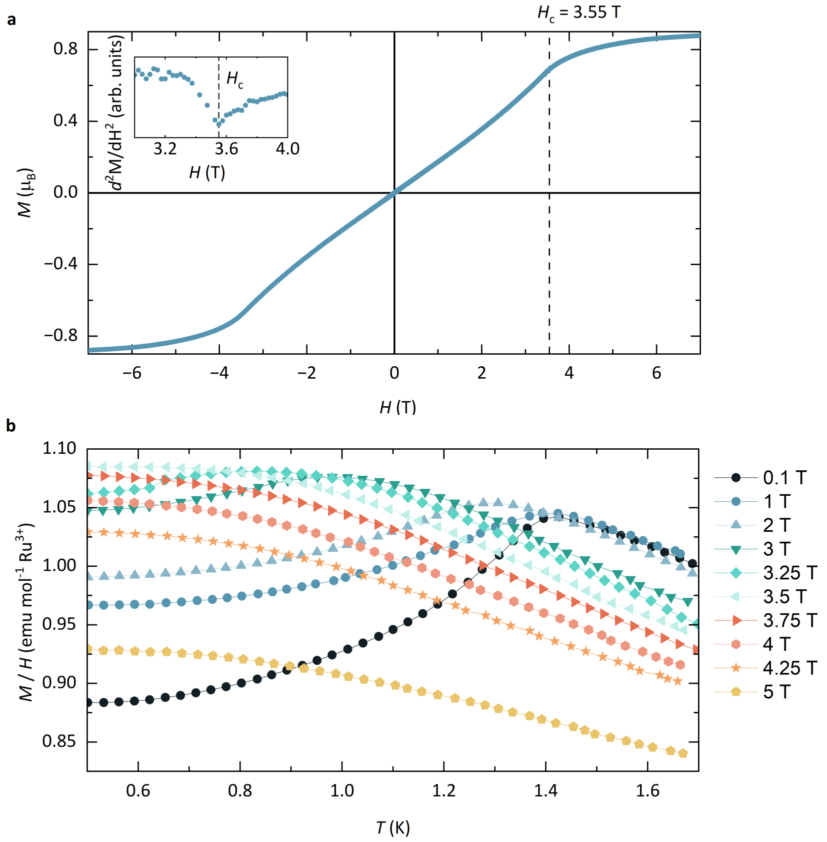

Supplementary Figure 2 **a** The magnetisation isotherm of RPSO measured at 0.4 K increases linearly with the applied field up to 2 T, beyond which the rate of increase in magnetisation becomes steeper before reaching a critical field, $H_\mathrm{C} = 3.55$ T. **a, inset** The second field derivative of the magnetisation highlighting $H_\mathrm{C} = 3.55$ T. **b** The effect of this critical field can be observed in the field dependence of M/H, where the ordering temperature is suppressed above H_C.

the on-site Coulomb repulsion $U = 3$ eV and Hund's coupling $J_\mathrm{H} = 0.46$ eV, the latter value determined by RIXS.

To validate the experimental observation of the $j_\mathrm{eff} = \frac{1}{2}$ state of Ru^{3+} in RPSO and to directly compare it with α-$RuCl_3$, we have performed electronic band structure calculations for both systems at the scalar relativistic level (see Supplementary Figure 3). To ensure the validity of this comparison, we employed the widely accepted low-temperature rhombohedral structure for band-structure calculations of α-$RuCl_3$ [7]. In this $R\bar{3}$ structure, the octahedral crystal field is trigonally distorted as in RPSO, yielding the same local 3-fold rotational symmetry at the Ru^{3+} sites of each system. Calculating the density of states allows the orbital energies of each system to be determined from their Wannier projections, and the corresponding octahedral crystal field splitting, Δ_O, for RPSO was calculated to be 2.52 eV, compared with 2.42 eV for α-$RuCl_3$. The increased Δ_O for the oxide crystal field of RPSO compared with the chloride crystal field of α-$RuCl_3$ is in keeping with the RIXS data analysis and reflects the greater ionic character

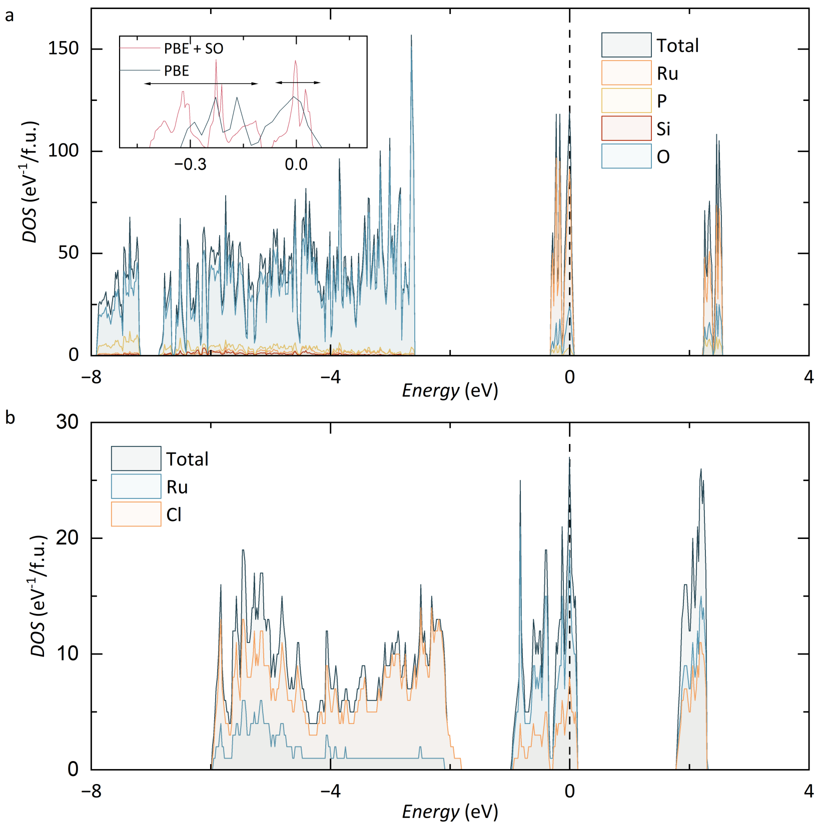

Supplementary Figure 3 PBE density of states for **a** RPSO and **b** α-$RuCl_3$ (bottom). The Fermi level (E_f) is at zero energy. The inset shows a comparison of PBE and PBE+SO density of states around E_f, with the arrows labeling the regions of $j_{eff} = \frac{3}{2}$ and $\frac{1}{2}$ states.

of O^{2-} versus Cl^-. The trigonal crystal field splitting, on the other hand, is equivalent in magnitude but opposite in sign in both systems with $|\Delta_t| = 0.06$ eV. Overall, the calculated orbital energies indicate that RPSO meets the same prerequisite requirements for the formation of the $j_{eff} = \frac{1}{2}$ state for Ru^{3+} as in α-$RuCl_3$, which is fully consistent with the RIXS data analysis in the main manuscript.

This conclusion is verified by calculating the PBE+SO density of states of RPSO. The resulting density of states reveals a clear splitting of the t_{2g} band into lower and higher lying $j_{eff} = \frac{1}{2}$ and $j_{eff} = \frac{3}{2}$ manifolds, respectively, as has also been seen in the PBE+SO density of states of Ir^{4+} compounds [8]. Spin-polarised calculations (PBE+U+SO) further reveal that the magnetic moments of Ru^{3+} comprise $\mu^x_{spin} = 0.19\ \mu_B$ of the spin contribution and $\mu^x_{orb} = 0.38\ \mu_B$ of the orbital contribution when directed along a. Similarly, along the c axis, the magnetic moments are $\mu^z_{spin} = 0.20\ \mu_B$ and $\mu^z_{orb} = 0.47\ \mu_B$. The almost isotropic nature of the moment and the nearly $1:2$ ratio of μ_{spin} and μ_{orb} are both consistent with a $j_{eff} = \frac{1}{2}$ state in RPSO.

We also expanded our DFT calculations to include the magnetic exchange interactions between the $j_{eff} = \frac{1}{2}$ states of the Ru^{3+} ions in RPSO by analysing the hopping integrals t within superexchange theory [9, 10]. Using $U = 3$ eV, as well as $J_H = 0.46$ eV and $\lambda = 0.13$ eV from RIXS, we find $(J, K, \Gamma, \Gamma') = (-0.16, -0.65, 0.26, -0.42)$ meV along with $\zeta = 0.03$ meV and $\xi = 0.02$ meV. These K and Γ values are compatible with the LSWT estimates, whereas Γ' seems overestimated, and J is weakly ferromagnetic, in contrast to the weakly antiferromagnetic J required for stabilising the Néel order. These discrepancies may be caused by the overall weakness of the exchange couplings and by the residual trigonal crystal-field splitting, which was not taken into account within the superexchange theory. Nevertheless, these estimates support our description of RPSO in terms of the $\mathcal{H}_{JK\Gamma\Gamma'}$ model and justify the neglect of ζ and ξ as minor terms in the spin Hamiltonian. We also estimated the interlayer coupling of $J_\perp = 0.18$ meV, which is somewhat weaker than the leading in-plane terms. Our calculations confirm that further-neighbor in-plane couplings should be as small as $J_2 \approx 0.003$ meV and $J_3 \approx 0.0006$ meV and thus truly negligible in this material. Finally, we note that while the Ru-Ru superexchange pathway in RPSO does not directly map to the planar geometry originally proposed in the Jackeli-Khaliullin mechanism [11], no chemical compound studied to date as a Kitaev QSL candidate has shown the exact cancellation of the hopping matrix elements required for the pure Kitaev model [9, 12]. For RPSO,

Supplementary Table 3 Exchange parameters (in meV) of the five lowest χ^2 solutions of the $\mathcal{H}_{XXZ}$ model obtained for RPSO in this work by fitting inelastic neutron scattering data to linear spin wave theory.

RPSO	J^{xy}	J^{z}	$J^{\pm\pm}$	$J^{z\pm}$	χ^2
Solution 1	0.08	0.28	−0.10	−0.52	67.7
Solution 2	0.05	0.28	−0.10	−0.36	68.9
Solution 3	0.02	0.28	−0.10	−0.25	69.7
Solution 4	0.07	0.27	−0.10	−0.42	69.7
Solution 5	0.08	0.29	−0.10	−0.61	70.5

the hopping parameters, as defined in Ref. [9], are found to be $t_2 = 0.054$ meV, which is approximately twice as large as $t_4 = 0.032$ meV, while t_1 and t_3 are both below 0.010 meV and, therefore, negligible. This confirms the proximity of RPSO to the ideal Jackeli-Khaliullin scenario despite the non-coplanar geometry of its superexchange pathway.

Supplementary Note 4: Inelastic Neutron Scattering Data Analysis

As shown in Supplementary Figure 4, at an incident energy of $E_\mathsf{i} = 3.14$ meV, the experimental dynamical structure factor shows only magnetic scattering. Above T_N, the $\Delta = 0.1$ meV gap closes, and the spectra are peaked at low $|Q|$ and show scattering up to $\Delta E \approx 0.8$ meV. This behavior is observed up to 15 K and is shown in Supplementary Figure 4, which includes the spectrum and representative cuts at selected temperatures.

As detailed in the main manuscript, a grid search was used to explore and compare the $\mathcal{H}_{JK\Gamma\Gamma'}$ parameter space to the experimental dynamical structure factor at 0.08 K. The resulting five-dimensional space, encompassing the fitted parameter sets of (J, K, Γ, Γ') and their corresponding goodness-of-fit χ^2 to the data, is shown in Supplementary Figures 5 and 6 for $K > 0$ and $K < 0$, respectively. Here, missing spaces in the four-dimensional spaces are solutions that are incompatible with the magnetic structure. A summary of the number of the lowest χ^2 parameter sets — subsequently used for the simulated annealing optimisation (see Methods) — is presented in Supplementary Figure 7. The best fitting optimised solutions for $K < 0$ are presented in Table 1 in the main text. We note that the mismatch in χ^2 values between the grid search and the simulated annealing optimisation stems from the different number of cuts used for fitting — 8 in the former and 60 in the latter.

For low χ^2 solutions with $K > 0$, we find a connection to the optimised $K < 0$ parameter sets through the self-duality of the $\mathcal{H}_{JK\Gamma\Gamma'}$ model when defined using the set of rotated cubic axes. Within this frame of reference, a physically identical set of parameters can be obtained by applying a π rotation to the honeycomb plane about the crystallographic c-axis [13]

$$\begin{pmatrix} J \\ K \\ \Gamma \\ \Gamma' \end{pmatrix}_{\mathsf{dual}} = \begin{pmatrix} 1 & \frac{4}{9} & -\frac{4}{9} & \frac{4}{9} \\ 0 & -\frac{1}{3} & \frac{4}{3} & -\frac{4}{3} \\ 0 & \frac{4}{9} & \frac{5}{9} & \frac{4}{9} \\ 0 & -\frac{2}{9} & \frac{2}{9} & \frac{7}{9} \end{pmatrix} \begin{pmatrix} J \\ K \\ \Gamma \\ \Gamma' \end{pmatrix}.$$

This transformation is evident for the representative optimised (J, K, Γ, Γ') parameter sets, $(-0.16, 0.86, -0.01, 0.12)$ meV and $(-0.11, 0.56, 0.12, 0.09)$ meV, with $\chi^2 = 68.8$ and 72.4 respectively. Applying the transformation matrix to these solutions yields $(0.28, -0.46, 0.43, -0.1)_{\mathsf{dual}}$ meV and $(0.12, -0.15, 0.36, -0.02)_{\mathsf{dual}}$ meV, corresponding approximately to Solutions 1 and 3 in Table 1.

A recent trend in the literature is a growing preference for the crystallographic frame to formulate the exchange Hamiltonian of the extended Kitaev model [13, 14]. In this framework, the exchange Hamiltonian is expressed as $\mathcal{H}_{XXZ}$ (Equation 3), and the optimised solutions in Table 1 can be rewritten using Equation 3, resulting in the parameters presented in Supplementary Table 3. Using this notation, only $J^{z\pm}$ varies across the the optimised solutions, while $(J^{xy}, J^{z}, J^{\pm\pm})$ are consistently around $(0.05, 0.28, -0.1)$ meV.

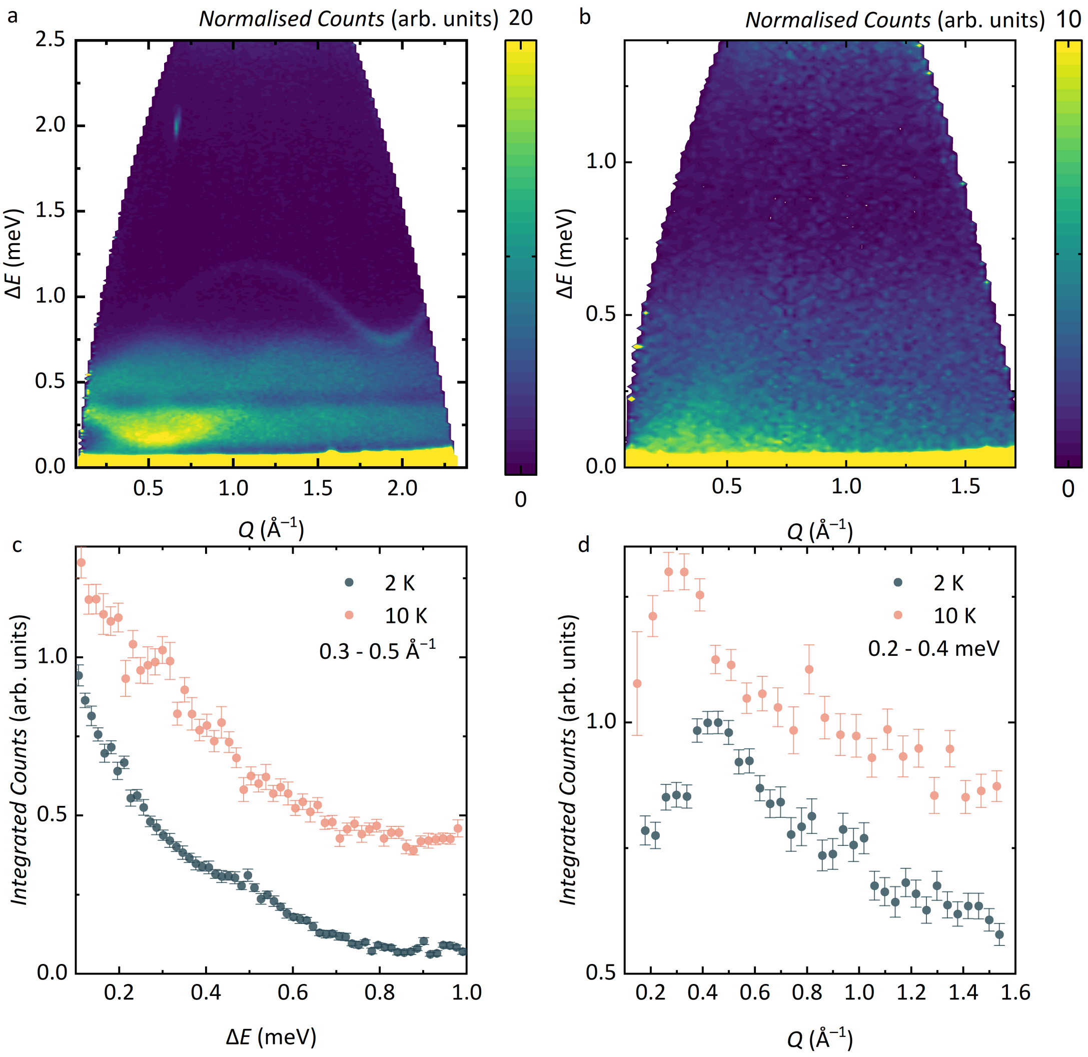

Supplementary Figure 4 **a** The experimental dynamical structure factor, $S(Q, \Delta E = \hbar\omega)_{\mathrm{exp}}$ of RPSO measured at 0.08 K and E_{i} = 3.14 meV (LET, ISIS) shows a clear excitation gap of approximately 0.1 meV and two branches of magnetic excitations extending up to 0.8 meV. **b** Above T_{N} at 2 K, the excitation gap closes, and **c-d** ΔE- and $|Q|$-integrated cuts show that structured magnetic scattering remains up to 10 K.

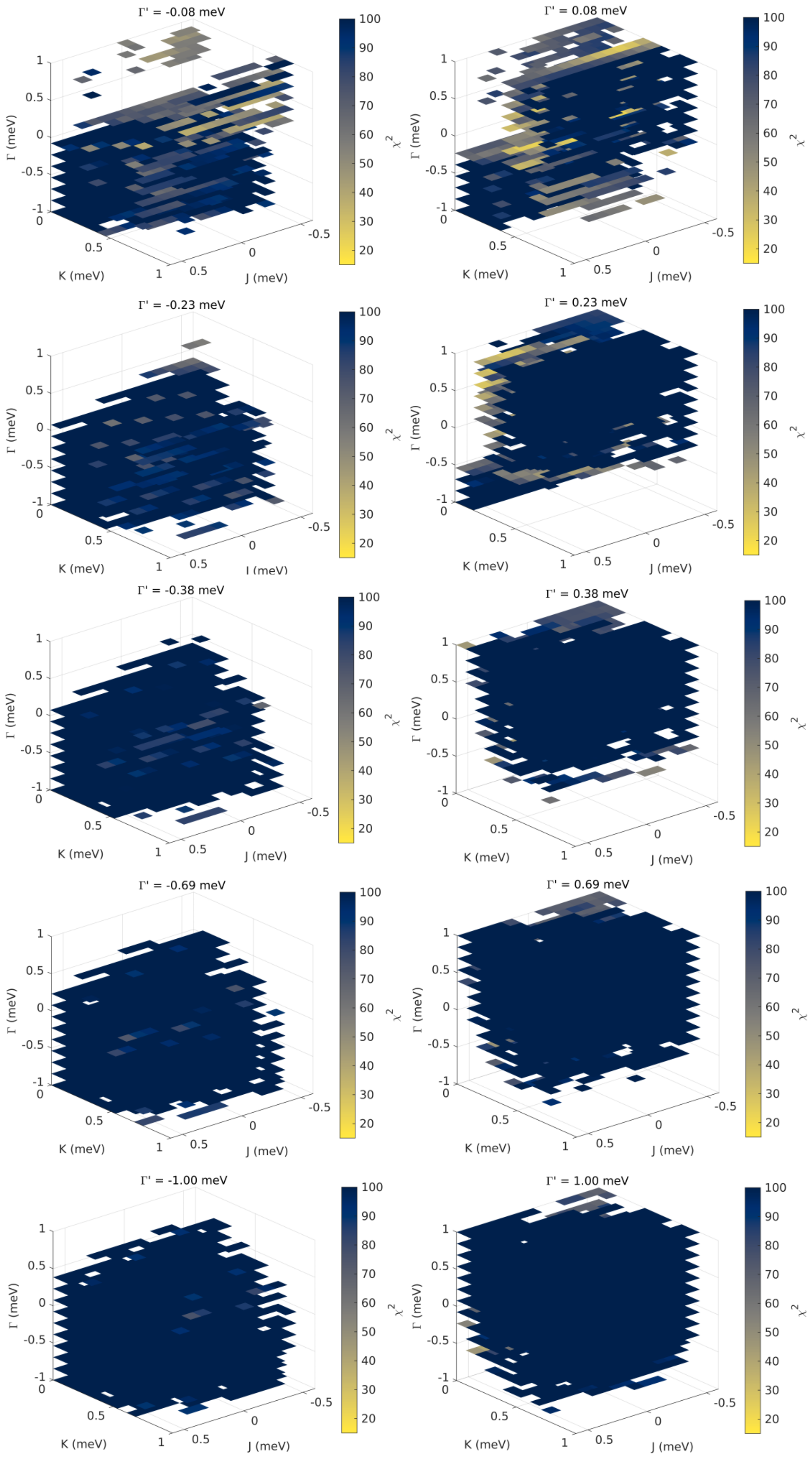

Supplementary Figure 5 Fit quality, represented by χ^2, for the $\mathcal{H}_{JK\Gamma\Gamma'}$ model fitted to 8 ΔE cuts with $K > 0$. Each four-dimensional space illustrates the variation of $-0.6 < J < 0.6$ meV, $0 < K < 1$ meV, and $-1 < \Gamma < 1$ meV, in 14 linearly spaced steps for a specific Γ' value, with the intensity reflecting the corresponding χ^2 values. Missing regions represent solutions that are incompatible with the Néel ordered ground state.

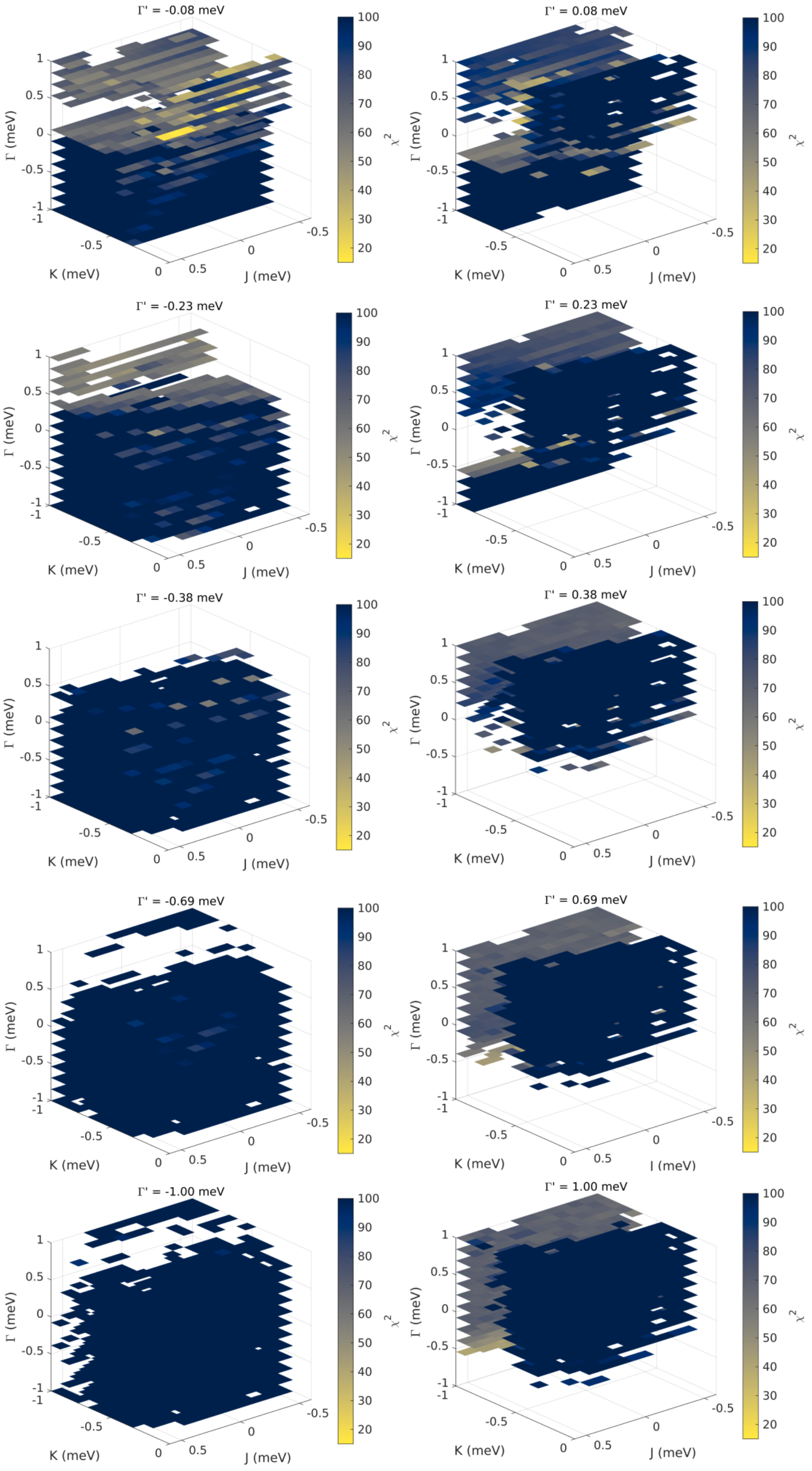

Supplementary Figure 6 Fit quality, represented by χ^2, for the $\mathcal{H}_{JK\Gamma\Gamma'}$ model fitted to 8 ΔE cuts with $K < 0$. Each four-dimensional space illustrates the variation of $-0.6 < J < 0.6$ meV, $-1 < K < 0$ meV, and $-1 < \Gamma < 1$ meV, in 14 linearly spaced steps for a specific Γ' value, with the intensity reflecting the corresponding χ^2 values. Missing regions represent solutions that are incompatible with the Néel ordered ground state.

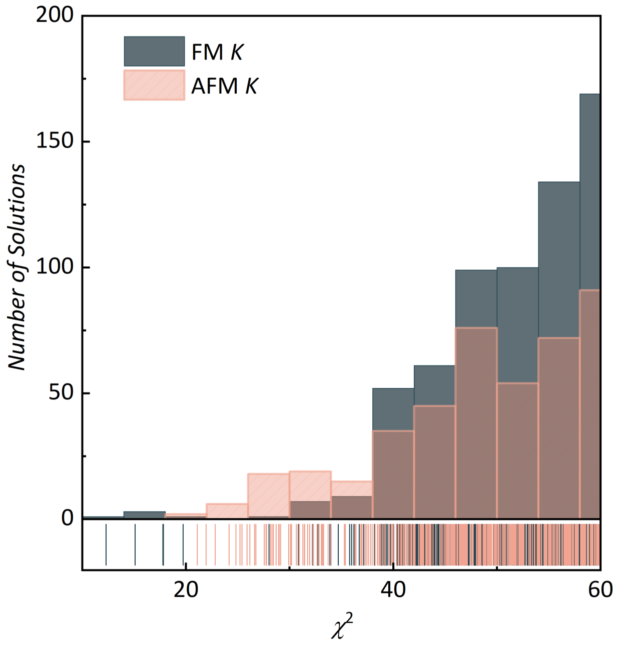

Supplementary Figure 7 The number solutions as a function of χ^2 for the parameter sets obtained in the $\mathcal{H}_{JK\Gamma\Gamma'}$ grid search for $K > 0$ and $K < 0$.

References

[1] Badrtdinov, D.I., Ding, L., Ritter, C., Hembacher, J., Ahmed, N., Skourski, Y., Tsirlin, A.A.: MoP_3SiO_{11} honeycomb antiferromagnet with disconnected octahedra. Phys. Rev. B **104**, 094428 (2021)

[2] Elbouaanani, L.K., Malaman, B., Gérardin, R.: Synthesis, crystal structure, and magnetic properties of FeP_3SiO_{11}: First example of iron(III)silicophosphate. J. Solid. State Chem. **147**, 565–572 (1999)

[3] Koepernik, K., Eschrig, H.: Full-potential nonorthogonal local-orbital minimum-basis band-structure scheme. Phys. Rev. B **59**, 1743–1757 (1999)

[4] Kresse, G., Furthmüller, J.: Efficiency of ab-initio total energy calculations for metals and semiconductors using a plane-wave basis set. Computational Materials Science **6**, 15–50 (1996)

[5] Kresse, G., Furthmüller, J.: Efficient iterative schemes for ab initio total-energy calculations using a plane-wave basis set. Phys. Rev. B **54**, 11169–11186 (1996)

[6] Perdew, J.P., Burke, K., Ernzerhof, M.: Generalized gradient approximation made simple. Phys. Rev. Lett. **77**, 3865–3868 (1996)

[7] Zhang, H., McGuire, M.A., May, A.F., Chao, H.-Y., Zheng, Q., Chi, M., Sales, B.C., Mandrus, D.G., Nagler, S.E., Miao, H., Ye, F., Yan, J.: Stacking disorder and thermal transport properties of α-$RuCl_3$. Phys. Rev. Mater. **8**, 014402 (2024)

[8] Bertinshaw, J., Kim, Y.K., Khaliullin, G., Kim, B.J.: Square lattice iridates. Ann. Rev. Condens. Matter Phys. **10**, 315–336 (2019)

[9] Winter, S.M., Li, Y., Jeschke, H.O., Valentí, R.: Challenges in design of Kitaev materials: Magnetic interactions from competing energy scales. Phys. Rev. B **93**, 214431 (2016)

[10] Rau, J.G., Lee, E.K.-H., Kee, H.-Y.: Generic spin model for the honeycomb iridates beyond the Kitaev limit. Phys. Rev. Lett. **112**, 077204 (2014)

[11] Jackeli, G., Khaliullin, G.: Mott insulators in the strong spin-orbit coupling limit: From Heisenberg to a quantum compass and Kitaev models. Phys. Rev. Lett. **102**, 017205 (2009)

[12] Rousochatzakis, I., Perkins, N., Luo, Q., Kee, H.-Y.: Beyond Kitaev physics in strong spin-orbit coupled magnets. Rep. Prog. Phys. (2024)

[13] Maksimov, P.A., Chernyshev, A.L.: Rethinking α-$RuCl_3$. Phys. Rev. Res. **2**, 033011 (2020)

[14] Halloran, T., Desrochers, F., Zhang, E.Z., Chen, T., Chern, L.E., Xu, Z., Winn, B., Graves-Brook, M., Stone, M.B., Kolesnikov, A.I., Qiu, Y., Zhong, R., Cava, R., Kim, Y.B., Broholm, C.: Geometrical frustration versus Kitaev interactions in $BaCo_2(AsO_4)_2$. Proc. Natl. Acad. Sci. U.S.A. **120**, 2215509119 (2023)
